# Pseudo attP sites in favor of transgene integration and expression in cultured porcine cells identified by streptomyces phage phiC31 integrase

**DOI:** 10.1186/1471-2199-14-20

**Published:** 2013-09-08

**Authors:** Yanzhen Bi, Ximei Liu, Long Zhang, Changwei Shao, Zhuo Ma, Zaidong Hua, Liping Zhang, Li Li, Wenjun Hua, Hongwei Xiao, Qingxin Wei, Xinmin Zheng

**Affiliations:** 1 Hubei Key Laboratory of Animal Embryo Engineering and Molecular Breeding, Institute of Animal Science and Veterinary Medicine, Hubei Academy of Agricultural Science, Wuhan 430064, China; 2 College of Animal Science and Technology, Yangtze University, Jingzhou 434025, China; 3 College of Life Science, Wuhan University, Wuhan 430072, China; 4 College of Animal Science and Technology, Northeast Agricultural University, Harbin 150030, China

**Keywords:** PhiC31 integrase, Pig, Pseudo attP site, TAIL-PCR

## Abstract

Phage PhiC31 integrase integrates attB-containing plasmid into pseudo attP site in eukaryotic genomes in a unidirectional site-specific manner and maintains robust transgene expression. Few studies, however, explore its potential in livestock. This study aims to discover the molecular basis of PhiC31 integrase-mediated site-specific recombination in pig cells. We show that PhiC31 integrase can mediate site-specific transgene integration into the genome of pig kidney PK15 cells. Intramolecular recombination in pig PK15 cell line occurred at maximum frequency of 82% with transiently transfected attB- and attP-containing plasmids. An optimal molar ratio of pCMV-Int to pEGFP-N1-attB at 5:1 was observed for maximum number of cell clones under drug selection. Four candidate pseudo attP sites were identified by TAIL-PCR from those cell clones with single-copy transgene integration. Two of them gave rise to higher integration frequency occurred at 33%. 5′ and 3′ junction PCR showed that transgene integration mediated by PhiC31 integrase was mono-allelic. Micro- deletion and insertion were observed by sequencing the integration border, indicating that double strand break was induced by the recombination. We then constructed rescue reporter plasmids by ABI-REC cloning of the four pseudo attP sites into pBCPB + plasmid. Transfection of these rescue plasmids and pCMV-Int resulted in expected intramolecular recombination between attB and pseudo attP sites. This proved that the endogenous pseudo attP sites were functional substrates for PhiC31 integrase-mediated site-specific recombination. Two pseudo attP sites maintained robust extracellular and intracellular EGFP expression. Alamar blue assay showed that transgene integration into these specific sites had little effect on cell proliferation. This is the first report to document the potential use of PhiC31 integrase to mediate site-specific recombination in pig cells. Our work established an ideal model to study the position effect of identical transgene located in diverse chromosomal contexts. These findings also form the basis for targeted pig genome engineering and may be used to produce genetically modified pigs for agricultural and biomedical uses.

## Background

Transgene technology holds promising applications in biomedicine and agriculture. Classical DNA pronuclear microinjection is a reliable tool to produce transgenic animals, but the inefficiency and uncontrollability of integration site and copy number are the major limitations [1]. Retrovirus and transposons enhance gene transfer efficiency and achieve single-copy transgenesis, but these methods integrate transgene throughout the genome [2-6]. As a result, the transgene is likely to disrupt endogenous gene structure. Also, it is still subjected to position effect that results in expression variation, even transgene silencing [7].

These problems can be overcome by targeting the transgene to a specific genomic site via homologous recombination (HR). However, it is usually labor-intensive and time-consuming due to the extremely low frequency of HR in mammalian cells [8]. Recently developed hybrid nucleases like ZFN, TALEn and Cas9 were believed to have a fair targeting efficiency, but it is still challenging to screen nucleases with high affinity and specificity [9,10]. Hence, molecular tools that introduce site-specific transgene integration with robust gene expression are necessary for precise transgenesis applications.

Phage integrases carry out irreversible and unidirectional recombination between attachment sites of phage and bacteria genomes, known as attB and attP sites, respectively [11]. Interestingly, these prokaryote-derived integrases also function in eukaryotic cells and has been used to target transgene to specific sites in fly, silkworm, mouse and rat [12-15]. These native docking sites in eukaryote is called pseudo attP site. PhiC31 integrase system has become a powerful tool to produce transgenic animals [16]. However, few studies have explored its potential to modify livestock genomes, except that specific pseudo attP sites have been identified in bovine genome [17-19]. In particular, as pig is an economically important and biomedically significant model animal, efficiently targeted genome engineering method is a necessity. In this study, we demonstrated usefulness of PhiC31 integrase to mediate site-specific transgene integration into pig pseudo attP sites. We also performed a functional rescue assay to reconstitute the recombination activity of pig pseudo attP sites. Our data indicate that pig pseudo attP sites are in favor of transgene expression. This work paves a new way to conduct targeted pig genome engineering.

## Results

### An optimal molar ratio of PhiC31 integrase to substrate is important to its activity in pig cell environment

To know if PhiC31 integrase system functions in pig cell environment, we used pCMV-Int and pBCPB^+^ plasmids to detect extra chromosomal intramolecular recombination. The two plasmids contain the required components of PhiC31 system: the integrase, attB and attP recognition substrates placed in direct orientation. In this context, PhiC31 integrase will catalyze the recombination between attP and attB site, deleting LacZ gene and yielding attL and attR hybrid sites. Forty-eight hours post transfection of the two plasmids into pig kidney PK15 cell line, a cell PCR assay was conducted to detect attL and attR sites with specific primers. Expected products were only obtained from the co-transfection of both the two plasmids. Neither pCMV-Int nor pBCPB^+^ produced these amplicons. We also titrated the molar ratio between enzyme and substrate. It showed that molar ratio of 5:1 (pCMV-Int: pBCPB^+^ = 5:1) resulted in maximum recombination efficiency 82%. The result suggested that the three components of PhiC31 integrase system were sufficient to induce site-specific intramolecular recombination in pig cells, implying that no cofactors were required for PhiC31 integrase to function in pig cells.

**Figure 1:**
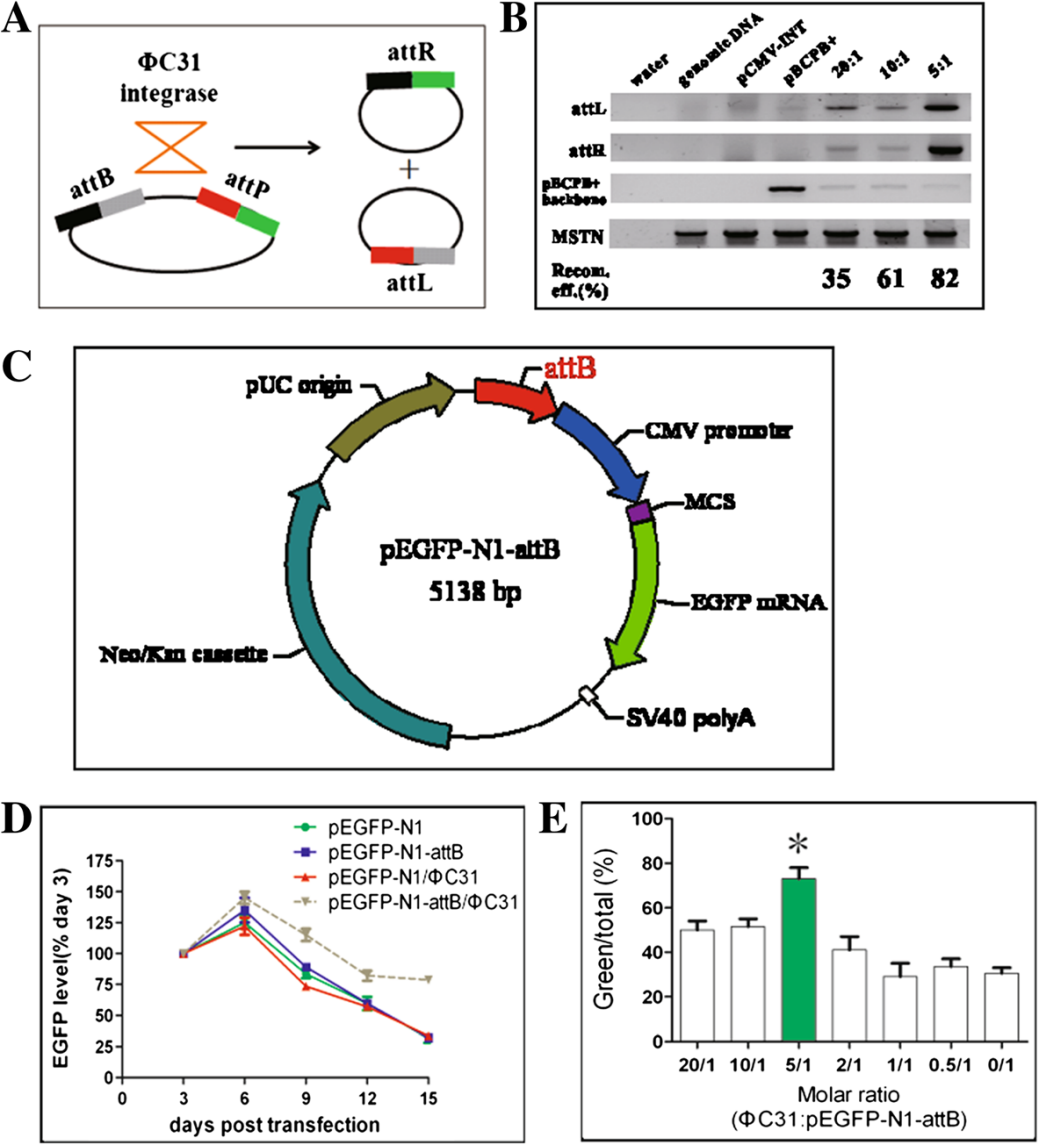
**PhiC31 integrase is active in pig cells. (A)** Mechanism of action of PhiC31 integrase-mediated site-specific recombination. The integrase catalyzes the intramolecular recombination between attB and attP sites within a single plasmid, producing two mini-circle plasmids with hybrid attR and attL sites, respectively. **(B)** PhiC31 integrase is functional in pig cells. Transfection of pig PK15 cells were performed with three different ratios of PhiC31 integrase plasmid to reporter pBCPB+. Resultant attL and attR sites were examined by PCR. The indicated recombination efficiency was calculated by measuring signal intensity in Visioncapture with the following formula: recombination efficiency = Intensity of attL/Intensity of (attL and pBCPB + backbone). A 5:1 ratio had 82% recombination efficiency. **(C)** The map of pEGFP-N1-attB (5138 bp). A 400 bp attB sequence was sub-cloned into the AseI site of pEGFP-N1, resulting in the site-specific integration reporter pEGFP-N1-attB. **(D)** EGFP expression level in a time lapse. The y-axis shows the expression levels and x-axis shows days after transfection. Four groups of plasmids (pEGFP-N1, pEGFP-N1-attB, pEGFP-N1/PhiC31 and pEGFP-N1-attB/PhiC31) were transfected into PK15 cells. EGFP expression level was quantified by measuring the extracellular EGFP by a Glomax Multi Jr fluorescence detector. IGF-I was detected by ELISA and used as an internal control for data normalization. pEGFP-N1-attB/PhiC31 maintained high EGFP level in comparison to other settings. **(E)** An optimal molar ratio of PhiC31 and pEGFP-C1-attB plasmids produced more cell clones expressing EGFP. Various ratios of PhiC31 and pEGFP-N1-attB plasmids were transfected into PK15 cells and cell clones were selected by G418. The y-axis shows % of green to total clones and x-axis shows different molar ratios. A 5:1 ratio produced more cell clones expressing EGFP, significant higher than other settings, including random integration (0/1 ratio). *, p < 0.01.

To test if PhiC31 integrase is capable of catalyzing intermolecular recombination, we constructed pEGFP-N1-attB (Figure 1C) and transfected it with pCMV-Int into PK15 cells. A time lapse assay was carried out to detect EGFP expression from day 3 to day 15. Co-transfection of pEGFP-N1-attB and pCMV-Int maintained high EGFP expression level when compared to other transfection groups. The pEGFP-N1 or pEGFP-N1-attB or pEGFP-N1 when used along with pCMV-Int, failed to maintain prolonged EGFP expression. This implied that PhiC31 integrase was likely to introduce intermolecular integration of attB-containing plasmid into certain locations of the genome of PK15 cells. Additional evidence was also obtained to verify site-specific intermolecular recombination. Cell clones were selected by transfecting pEGFP-N1-attB and pCMV-Int into PK15 cells at gradient molar ratios. After G148 selection of 7 days, we calculated the percentage of EGFP-positive clones to both EGFP-positive and G418-resistant colonies. In comparison to random integration of pEGFP-N1-attB, five transfection groups of both the two plasmids generated more number of cell clones. Interestingly, molar ratio at 5:1 of pCMV-Int to pEGFP-N1-attB generated maximum percentage of EGFP-positive clones, which is statistically significant than other transfection groups. This suggested that intermolecular recombination occurred between attB donor plasmid and pig chromosomes. Together, these data demonstrate that an optimal molar ratio of enzyme to substrate is critical to PhiC31 integrase system.

### Identification of candidate pig pseudo attP sites

**Figure 2:**
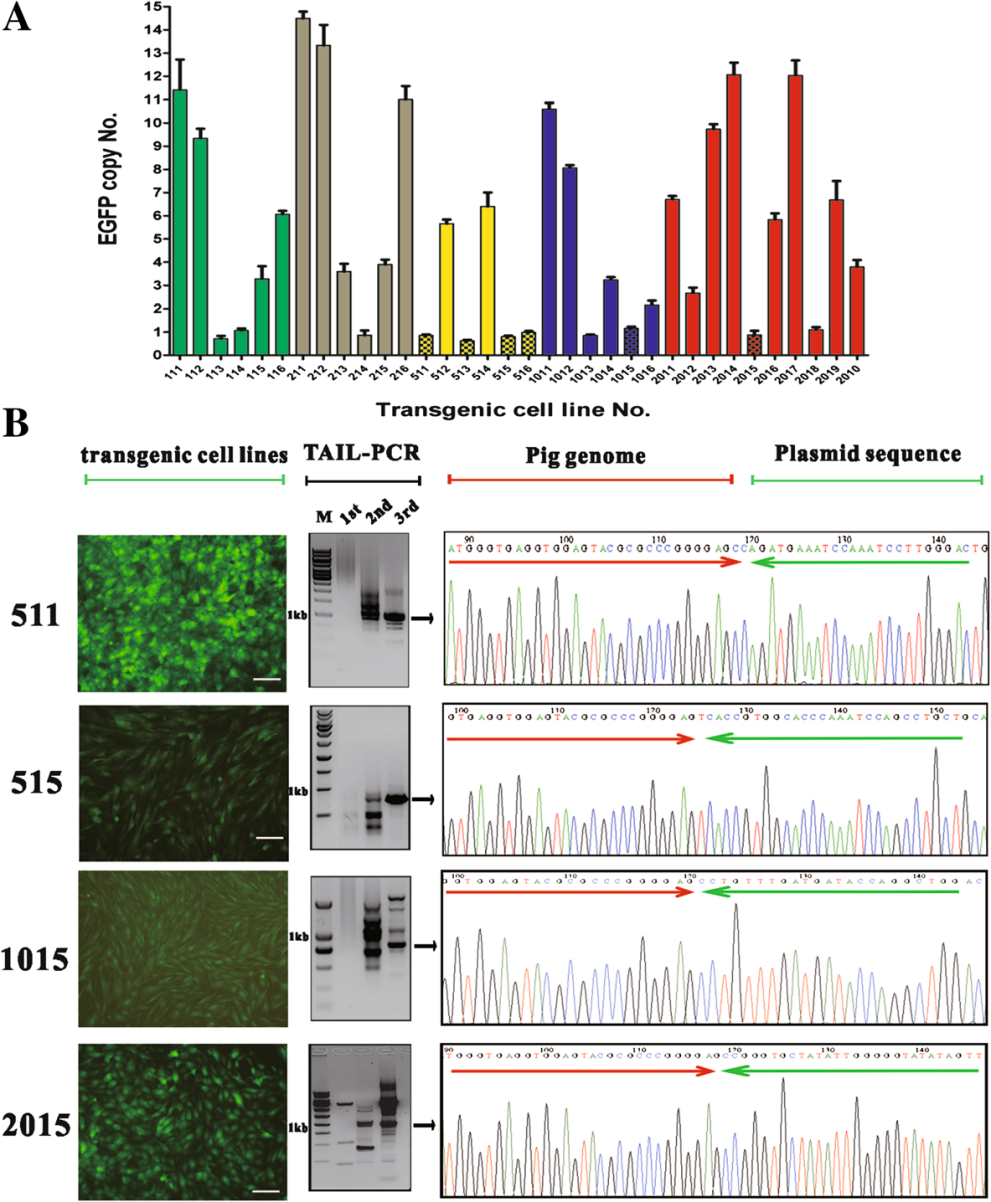
**Isolation of pig pseudo attP sites. (A)** Screening of transgenic cell lines with single-copy transgene integration by quantitative PCR. 34 transgenic cell lines expressing EGFP (shown in x-axis) were screened for single-copy transgene integration within PK15 cellular genome. This assay demonstrated that 11 cell lines harbored a single-copy pEGFP-N1-attB. The other 23 cell lines had distinct copy numbers of pEGFP-C1-attB, varying from 2 copies to 15 copies. **(B)** Isolation of integration sites. Left, microscopic images of four transgenic cell clones under fluorescence field (10×). Middle, TAIL-PCR was performed to isolate the integration sites. Right, Specific PCR products were TA cloned, sequenced and aligned by BALT against pig genome (UCSC). Red arrow represents pig genome sequence, and green arrow represents pEGFP-N1-attB sequence. The lanes shown as 1st, 2nd and 3rd represent the first, second and third round TAIL-PCR products fractionized by 1.5% agarose gel. M is 1 kb DNA molecular marker (Fermentas).

Next, we sought to identify the integration sites of reporter plasmid in the genome of PK15 cells. First, we used quantitative real-time PCR to screen single-copy transgene integrated cell clones. This manipulation not only allowed us to identify the precise location of candidate pseudo attP site but also to compare characteristics of the integration sites. Total number of 34 cell clones was picked from the transfection groups of different molar ratios. From these, eleven single-copy transgene integrated cell clones were identified, where the single-copy transgene integration frequency among G418-resistant cell clones is 33%. TAIL-PCR was then performed to isolate the integration sites [20]. As shown in Figure 2B (middle part), the size of TAIL-PCR products decreased round by round. Specific and sharp bands in the third round PCR were produced from four transgenic cell lines. TA cloning and Sanger sequencing of these specific bands revealed the sequence switch from attB to attR. The conversion from attB site to native PK15 cellular genome sequence near or at the TTG crossover and the readily detectable sequence similarity between genomic sequence and attP sites confirmed that the integration was induced by PhiC31 integrase. By aligning the native PK15 cellular genome sequence to UCSC Pig Genome database (SCGC Sscrofa10.2/susScr3), we retrieved four candidate pseudo attP sites, i.e. 5113, 5156, 1015, 2015 (Table 1). 5113 and 5156 pseudo attP sites were present in two cell clones, respectively. The integration frequency was 33% for the two pseudo sites when calculated in the 5:1 molar ratio group. 1015 and 2015 pseudo attP sites were identified from one cell clone, respectively. These four pseudo attP sites were located in four chromosomes. 5113 had minimum similarity of 18% to wild type attP site, while 2015 had a maximum similarity of 31%. No pseudo attP sites were isolated from the transfection groups of 1:1 and 2:1 molar ratios. This result again demonstrated that the molar ratio of enzyme to substrate was critical to the plasmid-chromosome recombination, reminiscent of the results of time lapse assay.

**Table 1:** Pseudo attP sites in pig kidney PK15 cell line

**Site**	**Sequence**^**a,b**^	**Identity to attP (%)**^**c**^	**Genomic location**	**Integration frequency**^**d**^
attP	CCCCAACTGGGGTAACCT**TTG**AGTTCTCTCAGTTGGGGG	100		
5113	AAGG*A*TT*TGG*AT*T*TCATC **TGT** GTA*T*GCTCAGTACTTTTT	18	Chr1, ﹢ , 114220089–114220127,Int.G^e^	2/6 = 33%
5156	TGGATTTG*GG*T*G*CC*AC*GG **TGA** CTCAGATGGGA*T*GCC*GGG*	26	Chr7, ﹣ , 45581124–45581162,Int.G	2/6 = 33%
1015	G*CC*TGGTATCATC*AA*ACA **TTT***A*TGGGATCTCTGCCAT*G*T	23	Chr9, ﹣ , 42746366–42746404,Int.G	1/6 = 17%
2015	ATA*C*CC*C*CAATA*TA*G*C*TC **TGA***A*A*T*A*C*CAATG*G*G*TG*TTCC	31	Chr3, ﹣ , 54698718–54698756,Int.G	1/6 = 17%

^a^The TTG core in the wild-type attP site and similar core in pseudo attP site of pig genome are shown in bold.

^b^Nucleotides identical to the wild-type site at that position are underlined.

^c^The identity is calculated by Bioedit.

^d^Integration frequency = recombination clones/total selected clones × 100%.

^e^Int.G, intergenic region.

**Figure 3:**
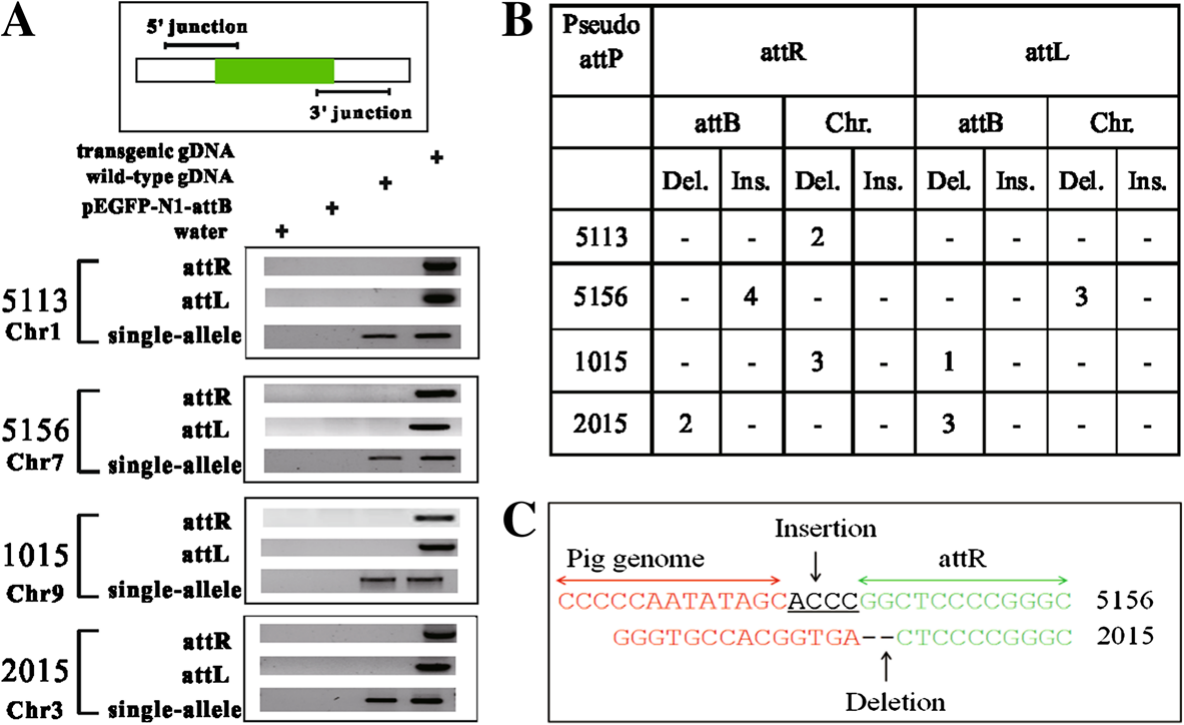
**Confirmation of integration sites and allele analysis. (A)** attR and attL detection by junction PCR. Integration site and its neighboring sequences were retrieved from UCSC Pig Genome. Primers were designed across the junction site. PCR analysis revealed that pEGFP-N1-attB had been integrated into the sites identified by TAIL-PCR. In addition, all the 4 integration events occurred to one chromosome as demonstrated, implying that the transgene is integrated as single-allele at the 4 pseudo attP sites. **(B)** Micro insertion or deletion at integration site. PCR products in Figure 3A were TA cloned and sequenced to analyze the integration fidelity. Micro insertion and deletion were observed in the integration sites, implying that DNA strand breakage and repair occurred during recombination. **(C)** Representative examples of micro insertion or deletion at integration site. For attR hybrid sequence of 5156 pseudo attP site, four nucleotides “ACCC” were inserted between PK15 cellular genome and transgene sequence. For attR of 2015 pseudo attP site, two nucleotides of attB were lost.

In order to prove the integrity of site-specific recombination, junction PCR was carried out to detect the specificity of DNA sequence comprised of pig genome and reporter plasmid. A specific DNA product was detected for both attL and attR sequences of all the four pseudo sites (Figure 3A). This assay also allowed us to discriminate the integration status at each allele. Junction PCR demonstrated that reporter plasmid was integrated into one allele of the pseudo attP sites, implying that PhiC31 integrase catalyzes mono-allelic transgene integration in pig cells.

Sequencing of the TAIL-PCR products further revealed the covalent linkage of reporter plasmid in PK15 cellular genome. The joining sites were in accordance with PhiC31 integrase-mediated attB and attP crossover reaction. It implies that PhiC31 integrase functions in pig cells to introduce attB-containing plasmid into pseudo attP sites. Meanwhile, we observed imperfect repairing in the junction sites (Figure 3B). For example, in the 5156 pseudo attP site, the recombination brought a micro insertion of 4 nucleotides between PK15 cellular genome and attR site. Micro deletion of 2 nucleotides occurred at the recombination site of 2015 pseudo attP site (Figure 3C). In Figure 3B, it was shown that micro mutations could occurr at attL or attR sites. We did not find large mutation as reported previously [21].

### Functional rescue assay proved that candidate pig pseudo attP sites were *bona fide* recombination sites

**Figure 4:**
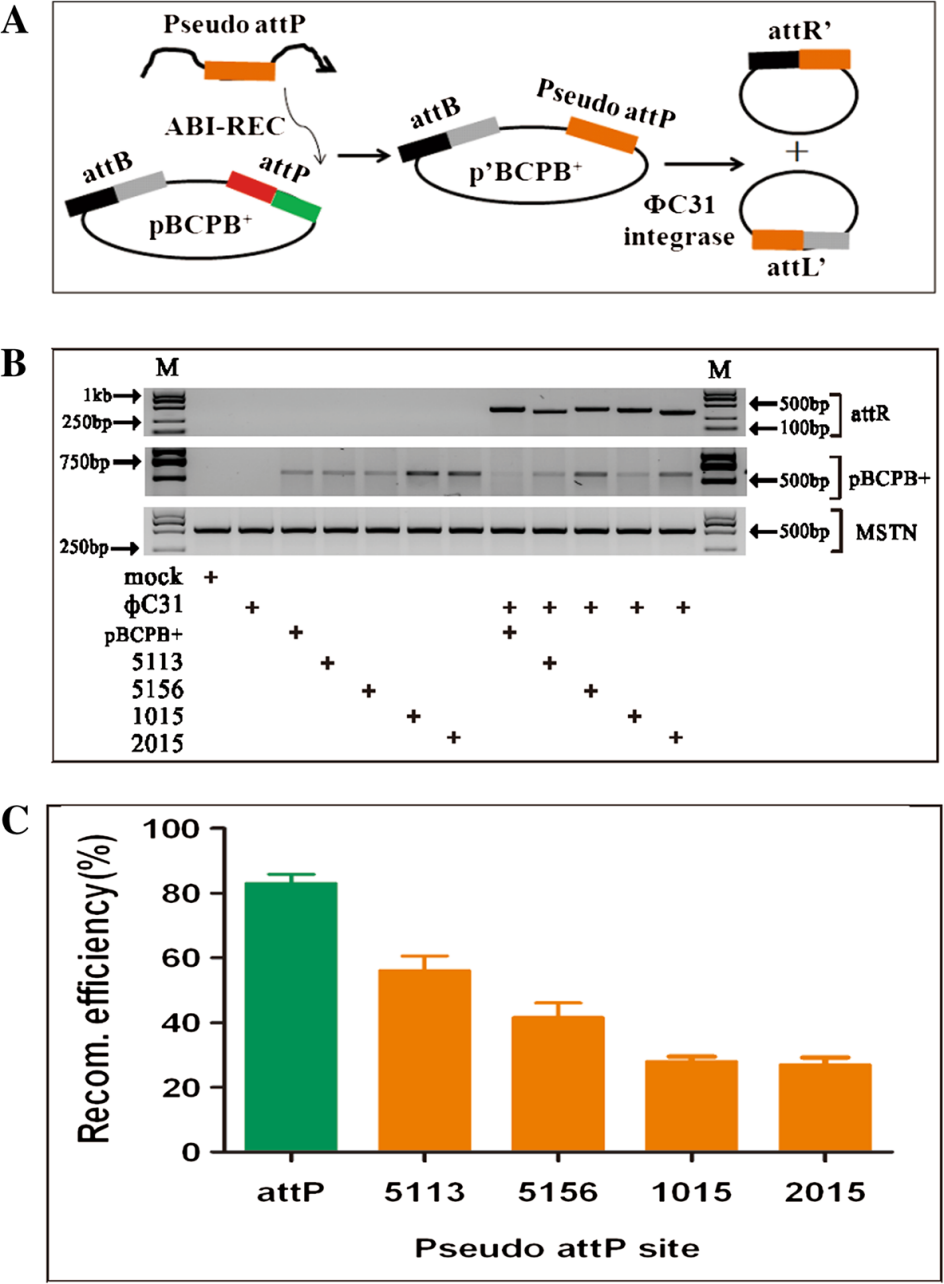
**Candidate pig pseudo attP sites can reconstitute site-specific recombination in a functional rescue assay. (A)** Scheme of reporter plasmid construction. Pig pseudo attP sites were cloned into pBCPB^+^ plasmid to replace the wild-type attP site by ABI-REC. The resultant plasmid was named p’BCPB^+^. This resulted in 4 p’BCPB^+^ plasmids, i.e. 5113, 5156, 1015, and 2015, respectively. p’BCPB^+^ was used to reconstitute the attB-pseudo attP recombination with the presence of PhiC31 integrase. For the ABI-REC details, please refer to additional files. **(B)** Rescue assay. PhiC31 integrase plasmid, pBCPB^+^, and 4 p’BCPB^+^ were transfected into PK15 cells. 48 h post transfection, genomic DNA was extracted and used for PCR detection of attR hybrid site with attR-F and attR-R primers. Pig endogenous MSTN gene (500 bp) was used as internal control to normalize the quantity of genomic DNA template. Primers attL-F and attR-R (555 bp) were used to quantify the amount of pBCPB^+^. **(C)** Recombination efficiency. The VisionCapture tool was used to quantify the density of the PCR product in (B). Recombination efficiency was calculated with the following formulation: Amount of attR/(amount of attR + amount of pBCPB^+^). It shows that the recombination efficiency of wild-type attP and attB sites was up to 80%. Pig 5113 pseudo attP site has a recombination efficiency of 60%, higher than the other 3 pig pseudo attP sites.

After identification of candidate pseudo attP sites in PK15 cellular genome, we then asked if they were functional to reconstitute site-specific recombination in vitro. To this end, we created reporter plasmid by cloning pig pseudo attP sites into pBCPB^+^ plasmid, replacing wild-type attP sequence. As restriction sites were unavailable besides wild-type attP site, it was achieved by ABI-REC cloning method (Figure 4A) [22]. The details of ABI-REC were shown in additional files (see Additional file 1: Figure S1, Additional file 2: Figure S2 and Additional file 3: FigureS3). This generated four reporter plasmids containing wild-type attB and pig pseudo attP sites that were in identical orientation. We hypothesized that if pig pseudo attP sites were active, PhiC31 integrase would recombine it with wild-type attB sequence to produce hybrid attL’ and attR’ sites. All these reporter plasmids were transfected into PK15 cell with pCMV-Int and junction PCR was carried out to detect the hybrid sites with specific primers. Specific DNA products were obtained for all the four pseudo attP sites (Figure 4B). As quantified by band density, we calculated the recombination efficiency. 5113 pseudo attP site mediated recombination at the efficiency of 58%, more active than the other three pseudo attP sites, but lower than wild-type attP site (Figure 4C). This assay proved that the four candidate pig pseudo attP sites were *bona fide* attP site that can induce site-specific recombination.

### Pig pseudo attP sites are in favor of robust transgene expression

**Figure 5:**
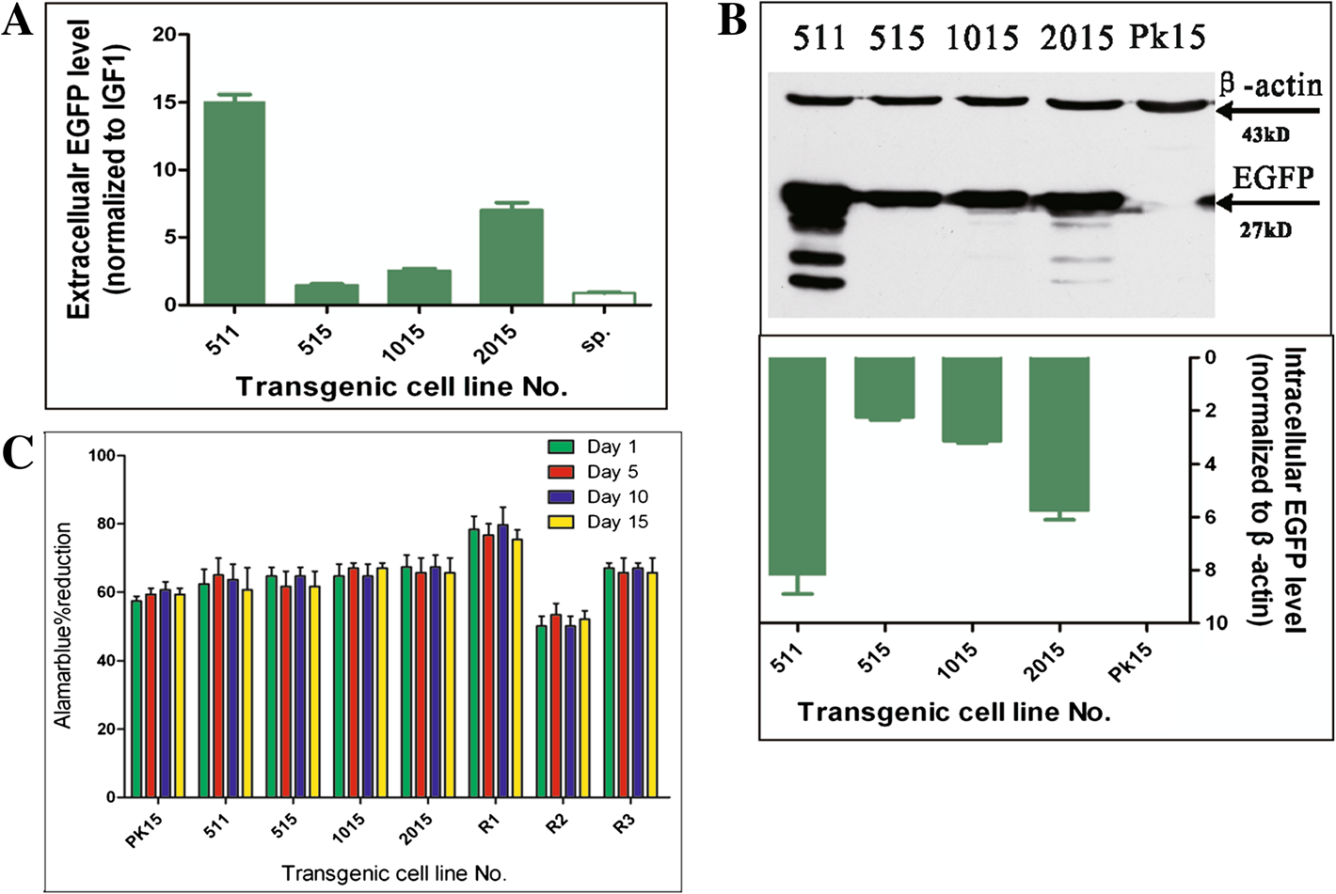
**Pig pseudo attP sites are in favor of robust transgene expression. (A)** Extracellular EGFP expression. Medium containing extracellular EGFP was harvested for EGFP fluorescence and IGF-I detection. IGF-I was internal control for input normalization. IGF-I concentration was measured by a porcine ELISA kit. Sp, the supernatant of PK15 cell line as negative control. **(B)** Intracellular EGFP level by western blot. Transgenic cells were harvested and quantified by a BCA protein quantification kit. Anti-EGFP antibody was used to detect the intracellular EGFP expression, and anti-β-actin antibody was used as internal control. MW of EGFP and pig β-actin is 27 kD and 43 kD, respectively. They are shown by arrows. PK15 cell lysate was used as negative control. The intensity of the bands was quantified by VisionCapture and converted into Prism to create the bar graph. **(C)** Cell proliferation assay. 10^3^ cells were seeded in a 96-well plate in triplicate. 10 μl Alamarblue indicator (Gibco) was added into the medium (with a final concentration of 10% v/v). The plate was incubated for additional 5 hours. The optical densities were measured at 570 nm and 630 nm in a micro-plate reader. Reduction percentage was calculated according to the formula provided in the instruction. Such a procedure was repeated in continuous 15 days. The reduction of four time points (day1, day5, day10 and day15) was indicated. Fresh medium was used as blank control. PK15 is the wild-type cell line. R1, R2 and R3 are randomly integrated transgenic cell lines.

We evaluated the favorability of the four pseudo attP sites to transgene expression. Extracellular EGFP expression was measured by a fluorescence detector. IGF-I was used as internal control to normalize the input variation as IGF-I was secreted into medium. It demonstrated that 5113 and 2015 pseudo attP sites were in favor of robust EGFP expression, significantly higher than that of 5156 and 1015 sites (Figure 5A). We then performed western blot to detect the intracellular EGFP level. As shown in Figure 5B, EGFP was efficiently expressed in these transgenic cell lines. Of these, 5113 and 2015 pseudo attP sites had higher EGFP level. Similar results were also observed under fluorescence microscopy, suggesting that extracellular and intracellular EGFP abundance were in consistent level. Thus, the two lines of evidence were mutually corroborated. Furthermore, an Alarmablue assay was conducted to measure the cell proliferation activity in a time lapse of 15 days. All the four transgenic cell lines exhibited consistent proliferation ability in comparison to wild-type PK15 cells and those with random integration. This indicated that transgene integration and expression into these sites had no adverse effect on cell proliferation ability and hence suggesting that these pseudo attP sites were in favor of transgene expression.

## Discussion

In this paper we demonstrated the catalyzing activity of PhiC31 integrase to introduce site-specific transgene integration into PK15 cellular genome. We showed that PhiC31 integrase system work synergistically in a quantitative manner. For the extra chromosomal site-specific recombination, it was found that the optimal ratio of pCMV-Int (6230bp) to pBCPB^+^ (7221bp) is 5:1 in pig cellular environment. PhiC31 integrase catalyzes the recombination as high as 82%. In an attempt to examine the potency of PhiC31 integrase-mediated intermolecular recombination, we also observed that a 5:1 molar ratio of pCMV-Int to pEGFP-N1-attB resulted in robust and long term EGFP expression and produced maximum percentage of EGFP-positive cell clones. Please note that in all the transfections we have assured the charge ratio of DNA plasmid to transfection reagent invariable. Hence, our results allow for a direct comparison among diverse transfection settings. Such results were also observed in previous reports [17,20]. Exact explanations of the “overproduction-inhibition effect” remain unknown. But careful examination of molar ratio of enzyme to substrate is a critical step prior to its successful use. This also suggests that, given that an unsuitable enzyme/substrate ratio is used, the efficacy of this system will be depressed.

Secondly, as documented previously in human, mouse, rat, fruit fly, silkworm and cattle, pseudo attP site profile varies across species [23]. Four pseudo attP sites were mapped in PK15 cellular genome in this work. All of them were located in intergenic regions, and none was found in other mammalian genomes. This indicates that PhiC31 integrase-mediated transgene integration pattern is species-specific, and the sites identified in this work are unique to pig. Our findings also suggest that pseudo attP sites in mammalian genome are not conserved during evolution. Additionally, this is unlike the integration pattern from virus gene transfer system, such as MLV that shows a strong preference for transcription start sequences [24]. In this regard, PhiC31 integrase functions in a safe way to modify host genome. On one hand, PhiC31 integrase is not likely to induce endogenous gene mutagenesis in pig cells. On the other hand, we did not observe any aberrant morphology and abnormal proliferation in the transgenic cell lines. Thus they are in favor of transgene integration and expression. Especially, 5113 and 5156 pseudo attP sites are ideal locus for robust transgene expression. This is particularly advantageous to gene therapy and safe transgenic technology, as this will reduce the risk of insertional mutagenesis [25,26].

Thirdly, we reconstituted the site-specific recombination by performing a functional rescue assay. To our knowledge, this is the first report to prove candidate pseudo attP site to be *bona fide* attP site. Through this test, we ruled out the possibility that pEGFP-N1-attB happened to be randomly integrated into candidate pig pseudo attP sites. This also indicates that the recognition site of PhiC31 integrase in PK15 cellular genome is not stringent, as the maximum similarity to wild type attP is around 30%. We observed an inverse correlation between similarity and recombination efficiency. It implies that there might be certain endogenous factors affect the sequence specificity of PhiC31 integrase [27]. But this needs a further research of binding proteins interacting with PhiC31 integrase. Hence, improvement of its recognition specificity is necessary in future studies to prevent aberrant recombination and multiple site integration [28].

Finally, we carried out TAIL-PCR to isolate the integration site. In previous studies, inverse PCR, half-nested PCR and plasmid rescue were often used to identify the candidate pseudo attP sites. But the common drawback of these methods is that they require a large starting amount of genomic DNA (usually 10 μg) for restriction and ligation. However, TAIL-PCR needs as little as 0.5 μg genomic DNA. In this work, we obtained four pig pseudo attP sites by TAIL-PCR. Moreover, the integration of reporter plasmid into all these four pseudo attP sites could be proved by junction PCR, indicating that the positive rate of TAIL-PCR is 100%. Accordingly, we believe that TAIL-PCR is a valuable tool owing to its ease, low cost and high reproducibility.

## Conclusions

Our data demonstrated that PhiC31 integrase functions efficiently for site-specific transgene integration into PK15 cellular genome. We isolated four pig pseudo attP sites and proved their recombination activity by a functional rescue assay. PhiC31 integrase induces mono-allelic transgene integration in pig cells. Pig pseudo attP sites were proved to be in favor of robust transgene expression. Our findings also established an ideal model to study the position effect of identical transgene structure located in diverse chromosomal contexts. These results form the basis for targeted pig genome engineering and may be used to produce genetically modified pigs for agricultural and biomedical uses.

## Methods

### Plasmid construction and DNA manipulation

The plasmid pCMV–Int (expressing PhiC31 integrase) and reporter plasmid pBCPB^+^ were gifts from M. P. Calos (Stanford University, USA). A Roche high pure PCR template preparation kit was used to extract genomic DNA. All plasmids were prepared by a TIANpure midi plasmid kit (Tiangen, Beijing, China). A Nanophotometer P-class was used to check the quality and quantity of DNA (Implen, Germany). AttB fragment was amplified from pBCPB^+^ and inserted into the AseI site of pEGFP-N1 (Clontech), and the resultant plasmid was pEGFP-N1-attB. For ABI-REC to create p’BCPB^+^ plasmids, pig pseudo attP sites were amplified by a KOD plus high-fidelity DNA polymerase and fused into pBCPB^+^ plasmid, replacing the wild-type attP site. For details of ABI-REC protocol, please refer to previous description [17]. Briefly, ABI-REC is a restriction-free DNA cloning method owning to asymmetric 3-primer PCR and intramolecular homologous recombination in bacteria. For extra chromosomal DNA recombination, we used a PCR assay with cell lysis to detect the hybrid sites. Briefly, 10^3^ cells were disrupted in lysis buffer (0.005% SDS and 1 mg/ml proteinase K) and incubated at 55°C for 1 hour and 94°C for 5 minutes. The lysis solution was centrifuged at 12000 rpm for 1 minute and the supernatant was transferred into a fresh tube. The supernatant contained chromosomal and extra chromosomal DNA and was used as template in a one-stop PCR [29]. Sequences of p’BCPB^+^ plasmids were supplemented in additional files. BglII, PstI, NdeI restriction nucleases (Fermentas, Lithuania) were used to checked the presence of pig pseudo attP sites.

### Cell culture, transfection and stable cell line selection

Pig kidney PK15 cell line was cultured in DMEM (Dulbecco’s modified Eagle’s medium, Gibco, Life Technologies) supplemented with 10% (v/v) FCS (fetal calf serum, Hyclone), 100 IU/ml penicillin and 0.1 mg/ml streptomycin. Cells were grown at 39°C in the presence of 5% CO_2_ and were subcultured (1:5) every 3 days. To obtain stable transfected cells, PK15 cells were split in a 35-mm plate until they reached 80% confluency. Cells were washed with PBS and then incubated with a mixture of 4 μg of DNA, 10 μl of Lipofectamine 2000 (Invitrogen) and 500 μl of Opti-MEM that was prepared as described by the manufacturer. To ensure the constant charge ratio of total nucleic acid and lipid, “filler or unrelated DNA” (pUC19 plasmid) was added to the transfection mixture. Following 6 h incubation, the medium was replaced with fresh medium. 48h post transfection, 600 μg/ml G418 (Sigma) was added. After 2 weeks of selection, G418-resistant colonies were picked manually and maintained in media with 50 μg/ml of G418. For cell proliferation assay, an Alamar blue kit (Gibco) was used. Briefly, 1000 cells were seeded in a 96-well plate in triplicate. 10 μl Alamar blue indicator was added into the medium (with a final concentration of 10% v/v). The plate was incubated for additional 5 hours. The optical densities were measured at 570 nm and 630 nm in a micro-plate reader (Multiskan MS; Labsystems, Helsinki, Finland). Reduction percentage was calculated according to the formula provided in the instruction (Gibco). Fresh medium was used as blank control. Such a procedure was repeatedly performed for continuous 15 days. The reduction values at day1, day5, day10 and day 15 were shown in the manuscript (Figure 5C).

### Extracellular EGFP quantification

Extracellular EGFP into medium was quantified using a Glomax Multi Jr fluorescence detector, according to the manufacture’s manual book. The EGFP level was indicated in arbitrary units. In order to normalize the input variation, an ELISA assay was used to measure the IGF-I expression level secreted into the medium as an internal control. Briefly, a 96-well plate was coated with anti-pig IGF-I antibody (Abcam, Cambridge, UK). After blocking, extracellular proteins were added in triplicate. IGF-I expression was then determined using a horseradish peroxidase-conjugated secondary antibody (Abcam) with 3, 3′, 5, 5′-tetramethylbenzidine (Sigma-Aldrich) as the substrate. The reaction was stopped by addition of 100 μL of 1 M H_2_SO_4_. Absorbance was measured at 450 nm using an ELISA reader (Bio-TEK Instruments Inc, Winooski, VT, USA).

### Western blot

The cells were washed thrice with ice-cold PBS to remove residual medium as much as possible. Afterwards the cells were lysed by NP40 Cell Lysis Buffer (Invitrogen) with phenylmethylsulfonyl fluoride (PMSF). After centrifugation, the supernatants were collected and quantified by Pierce BCA protein assay kit (Thermo Scientific). The total proteins were denatured by boiling for 5 minutes in a water bath and chilled in ice. 15% SDS-PAGE was used to size-fractionate the samples. Then they were transferred electrophoretically onto polyvinylidene difluoride membranes (Amersham Biosciences, Piscataway, NJ). After blocking with 2% milk, the membranes were incubated with primary antibodies at 4°C for 1 hour. The monoclonal primary antibody against EGFP (1:1000) was purchased from ProteinTech Co. Ltd. (Wuhan, China). The monoclonal primary antibody against β-actin (1:10000) was purchased from Sigma. After washing with PBST, the membranes were incubated with rabbit anti-mouse horseradish peroxidase-conjugated secondary antibodies (Sigma) for 2 h at room temperature. The signals were visualized by enhanced chemiluminescence (Perkin-Elmer, Norwalk, CT).

### Genome walking

Genome walking was carried out by a TAIL-PCR kit (Takara) according to the manufacture’s instruction. Briefly, 0.5 μg PK15 genomic DNA was used to perform the first round PCR with AP1, AP2, AP3, AP4 and IV-R1 primers in a 50 μl reaction, respectively. 1μl PCR products were used as template for 2nd and 3rd round PCR with IV-R2 and IV-R3 primers to obtain specific PCR bands. The DNA bands were excised and TA cloned into T vectors (Takara). Sanger sequencing was conducted in a ABI3730 DNA Analyzer (Applied Biosystems, Foster City, CA, U.S.) and the sequences were assembled and analyzed using DNAStar software (DNASTAR Inc., Madison, WI. U.S.). All primers are listed in Table 2.

**Table 2:** Primers used in this study

**Name**	**Sequence**	**Comments**
attB-F	GTCATTAATCGCCATTCAGGCTGCGCA	To amplify 400 bp attB site in AseI of pEGFP-N1
attB-R	GTCATTAATCTCGGCCTCGACTCTAG	
attL-F	GGCGAGAAAGGAAGGGAAG	To detect the attL site, 400 bp
attL-R	ATTAACCCTCACTAAAGGGA	
attR-F	TCAAAGTAAACGACATGGTG	To detect the attR site,520 bp
attR-R	CCAACCGCTGTTTGGTCTGC	
IV-R1	GCTTATAGATACCGTAGACAT	TAIL-PCR to identify integration site
IV-R2	TCCCGTGCTCACCGTGACC	
IV-R3	ATCAACTACCGCCACCTCG	
IV-F2	CGCCACCTCTGACTTGAGCG	
5113-LF	GTTGGGATTTGGCTCCAGCC	Use with IV-R1 for determination of 5′ end of 5113 pseudo attP site
5113-RR	TCTTAACACCCCATTGTTCTC	Use with IV-F2 for determination of 3′ end of 5113 pseudo attP site
5156-LF	GCCTTAATGAGGGCCAGAGC	Use with IV-R1 for determination of 5′ end of 5156 pseudo attP site
5156-RR	GTTGACCCATTTTCCTGCTCTTCG	Use with IV-F2 for determination of 3′ end of 5156 pseudo attP site
1015-LF	GTCCTGCCCTGACCCTTTGG	Use with IV-R1 for determination of 5′ end of 1015 pseudo attP site
1015-RR	GCCAACCTCCAAGTTGTTGG	Use with IV-F2 for determination of 3′ end of 1015 pseudo attP site
2015-LF	GCAAGGGGTT TTGAAACTGC	Use with IV-R1 for determination of 5′ end of 2015 pseudo attP site
2015-RR	AAAAGTGCCTCAGAACACCAC	Use with IV-F2 for determination of 3′ end of 2015 pseudo attP site
5113-pF	TCAAAGTAAACGACATGGTGGAGTGTAGCAGTTCCTAGGC	ABI-REC cloning of 5113 pseudo attP site in pBCPB^+^ plasmid
5113-pR	ATTAACCCTCACTAAAGGGATCTTAACACCCCATTGTTCTC	
5156-pF	TCAAAGTAAACGACATGGTGGACCTCTGCTGTGCCCAGAG	ABI-REC cloning of 5156 pseudo attP site in pBCPB^+^ plasmid
5156-pR	ATTAACCCTCACTAAAGGGACCAACCACTGGCTTCCCTCG	
1015-pF	TCAAAGTAAACGACATGGTGACCCCCACCCTGAGCTACTC	ABI-REC cloning of 1015 pseudo attP site in pBCPB^+^ plasmid
1015-pR	ATTAACCCTCACTAAAGGGAACTCCCCAGCAACCAGCTTC	
2015-pF	TCAAAGTAAACGACATGGTGATGAGACAAGAGGGACCAGG	ABI-REC cloning of 2015 pseudo attP site in pBCPB^+^ plasmid
2015-pR	ATTAACCCTCACTAAAGGGAAAAAGTGCCTCAGAACACCAC	
Pseudo-P1R	CACCATGTCGTTTACTTTGA	ABI-REC cloning of pig pseudo site
BCPB-S	GCTGCAAGGCGATTAAGTTGGG	Sequencing primer
EGFP-QF	TGAACCGCATCGAGCTGAAGGG	Real-time PCR primers, 130 bp
EGFP-QR	ACCTTGATGCCGTTCTTCTGCTTG	
TFRC-QF	GAGACAGAAACTTTCGAAGC	Real-time PCR primers, 81 bp
TFRC-QR	GAAGTCTGTGGTATCCAATCC	

### Real-time PCR

A Toyobo real-time SYBR Green I PCR master mix was used to amplify EGFP or TFRC gene in a Rotor Gene 6000 real-time rotary analyzer (Corbette Lifescience, Australia). The 20 μl PCR reaction mixture contained 10 μl 2 × master mix, 0.3 μl primer mix (5 μM each), 0.5 μl genomic DNA (50 ng) and 9.2 μl PCR-grade water. A two-step amplification protocol was used with the following parameters: 95°C for 2 minutes to pre-denature the template and activate Taq DNA polymerase followed by 40 cycles each of denaturation at 95°C for 8 seconds, annealing and extension at 60°C for 25 seconds. A final melting temperature analysis from 50°C to 99°C was used to ensure amplicon uniformity. Fluorescence was acquired at the step of annealing and extension. All PCR amplifications were performed in triplicate for each treatment. Wild-type PK15 genomic DNA was used as negative control. EGFP standard curve was produced by amplifying pEGFP-N1-attB at gradient concentration of 10 ng, 1 ng, 100 pg, 10 pg, 1 pg. TFRC standard curve was produced by amplifying wild-type gDNA at gradient concentration of 100 ng, 10 ng, 1 ng, 100 pg, 10 pg. Primers used for real-time PCR (Table 2) were designed and selected by Primer Premier 5. Relative signal intensities were calculated by the built-in software RG6000 series 1.7. Copy number of EGFP gene was calculated with the following formula: transgene copy number/pig genome = molecules of EGFP/molecules of TFRC × 2. TFRC is a single-copy gene in haploid pig genome.

### Statistical and bioinformatic analysis

The results of colony calculation, real-time PCR, EGFP expression were shown as the mean ± SD (n = 3). The statistical analysis was performed using *Student’s t-test* for comparison between two groups. The difference was considered significant if *p* < 0.05. PCR product quantification is carried out by VisionCapture [30]. The identity of pig pseudo attP site to wild-type attP site was determined using Bioedit.

## Abbreviations

TAIL-PCR: Thermal asymmetric interlaced PCR; ABI-REC: Asymmetric bridge PCR and intramolecular homologous recombination; EGFP: Enhanced green fluorescence protein; PBS: Phosphate-buffered saline; attB: Attachment site of bacteria; attP: Attachment site of phage; attL: Left attachment site; attR: Right attachment site; ELISA: Enzyme-linked immunosorbent assay; IGF-I: Insulin-like growth factor I; TFRC: Transferrin receptor.

## Competing interests

The authors declare that they have no competing interests.

## Authors’ contributions

YZ conceived the project, designed all the primers, analyzed all the experimental data and wrote the manuscript. XL performed cell culture, transfection and selection. LZ carried out TAIL-PCR. CS conducted immunoblotting. ZM performed ELISA assay. ZH performed TA cloning and sequence analysis. LZ performed PCR and real-time PCR. LL and WH were involved in plasmid preparation and keeping stocks. HX and QW performed cell proliferation assay. XZ proposed the functional rescue of pig pseudo attP site by ABI-REC method. All authors read and approved the final manuscript.

## Supplementary Material

Additional file 1: Figure S1 ABI-REC of pig pseudo attP sites into pBCPB^+^ plasmid. Fused products were indicated by arrow. M is molecular DNA ladder. 5113, 5156, 1015 and 2015 represents four pig pseudo attP sites cloned by ABI-REC. Click here for file

Additional file 2: Figure S2 p’BCPB^+^ plasmid restriction analysis. Recombinant plasmids were analyzed by restriction digestion. M is molecular DNA ladder. 5113, 5156, 1015 and 2015 stands for the four recombinant plasmids derived from backbone pBCPB^+^. For each plasmid, the first lane shows undigested DNA and the second lane shows the restricted DNA. Click here for file

Additional file 3: Figure S3 Partial sequences of four p’BCPB^+^ plasmids were shown. Native restriction site is underlined. Cloned pig pseudo attP site is shown in red, where the identified pseudo attP site is shown in bold. pBCPB^+^ plasmid sequence is shown in black. Recombination crossover is shown in bold and italics. Click here for file
