# Supplementary figures and images for: Pseudo attP sites in favor of transgene integration and expression in cultured porcine cells identified by streptomyces phage phiC31 integrase
Source: BMC Mol Biol. 2013 Sep 8;14:20. doi: 10.1186/1471-2199-14-20 (PMC3844521; doi:10.1186/1471-2199-14-20)

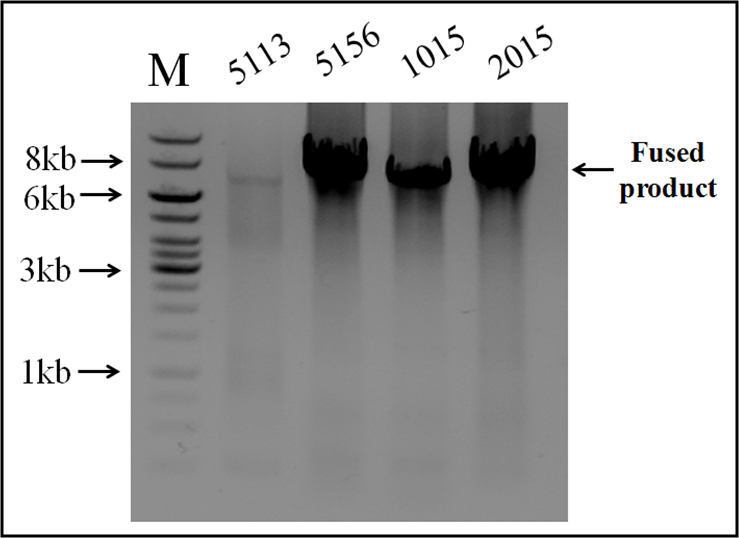

Supplement: Additional file 1: Figure S1 — ABI-REC of pig pseudo attP sites into pBCPB+ plasmid. Fused products were indicated by arrow. M is molecular DNA ladder. 5113, 5156, 1015 and 2015 represents four pig pseudo attP sites cloned by ABI-REC. [file 1471-2199-14-20-S1.tiff]

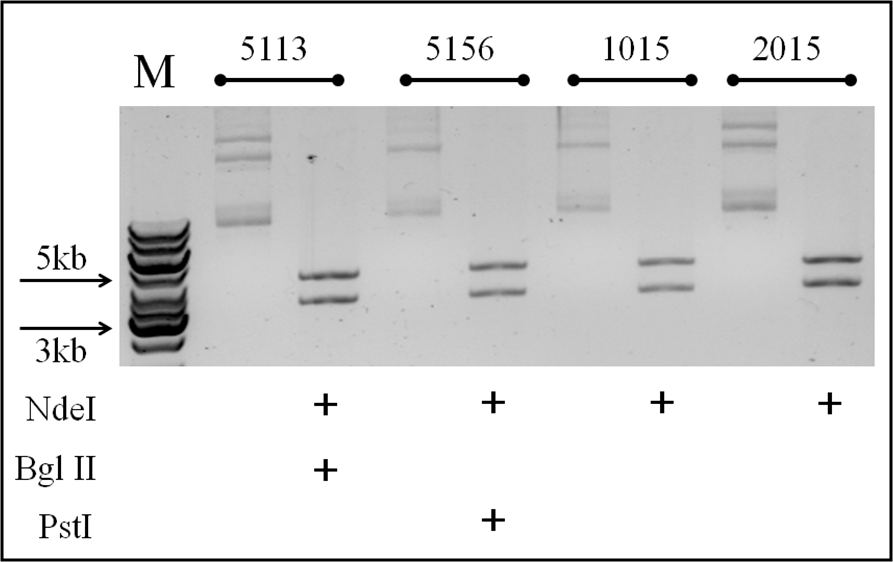

Supplement: Additional file 2: Figure S2 — p’BCPB+ plasmid restriction analysis. Recombinant plasmids were analyzed by restriction digestion. M is molecular DNA ladder. 5113, 5156, 1015 and 2015 stands for the four recombinant plasmids derived from backbone pBCPB+. For each plasmid, the first lane shows undigested DNA and the second lane shows the restricted DNA. [file 1471-2199-14-20-S2.tiff]

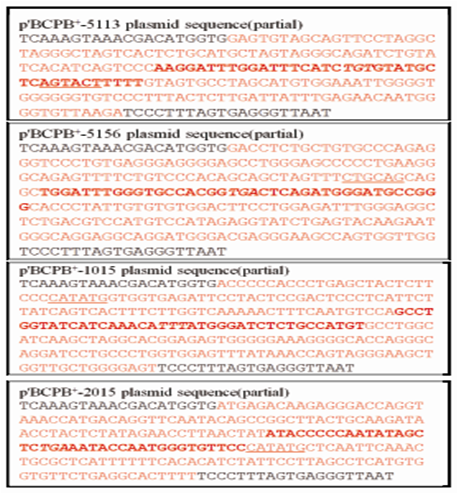

Supplement: Additional file 3: Figure S3 — Partial sequences of four p’BCPB+ plasmids were shown. Native restriction site is underlined. Cloned pig pseudo attP site is shown in red, where the identified pseudo attP site is shown in bold. pBCPB+ plasmid sequence is shown in black. Recombination crossover is shown in bold and italics. [file 1471-2199-14-20-S3.tiff]
